# Investigating the Mechanisms of AquaporinZ Reconstitution through Polymeric Vesicle Composition for a Biomimetic Membrane

**DOI:** 10.3390/polym12091944

**Published:** 2020-08-28

**Authors:** Danhua Zhou, Hu Zhou, Shufeng Zhou, Yen Wah Tong

**Affiliations:** 1 College of Chemical Engineering, Huaqiao University (Xiamen Campus), No.668 Jimei Avenue, Xiamen 361021, China; zhoudh@hqu.edu.cn (D.Z.); szhou@hqu.edu.cn (S.Z.); 2 NUS Environmental Research Institute (NERI), National University of Singapore (NUS), 1 Create Way, Singapore 138602, Singapore; zhouhusg@hotmail.com; 3 Department of Chemical and Biomolecular Engineering, National University of Singapore, Engineering Drive 4, Singapore 117576, Singapore

**Keywords:** AqpZ, polymeric vesicles, biomimetic membrane, composition-reconstitution

## Abstract

Aquaporin-Z (AqpZ) are water channel proteins with excellent water permeability and solute rejection properties. AqpZ can be reconstituted into vesicles utilizing cell-like bilayer membranes assembled from amphiphilic block copolymers, for the preparation of high-performance biomimetic membranes. However, only a few copolymers have been found suitable to act as the membrane matrix for protein reconstitution. Hence, this work analyzes the mechanism of protein reconstitution based on a composition-reconstitution relationship. The vesicle formation and AqpZ reconstitution processes in various amphiphilic block copolymers were investigated in terms of size, morphology, stability, polymeric bilayer membrane rigidity, and thermal behavior. Overall, this study contributes to the understanding of the composition-reconstitution relationship of biomimetic membranes based on AqpZ-reconstituted polymeric vesicles.

## 1. Introduction

Currently, more than 40% of the world’s population faces water shortages [1]. In the coming decades, water scarcity is anticipated to increase continuously due to population growth, climate change, urbanization, and increasing water consumption per capita [2]. Moreover, salt water constitutes 97.2% of Earth’s water, which offers a promising alternative source for fresh water. Hence, desalination technologies are attracting worldwide attention. One such potential desalination technology is Aquaporins (Aqps)-based biomimetic membrane water treatment, which promises high selectivity and low energy consumption [3,4,5,6,7]. AqpZ is a water channel protein with excellent water permeability and absolute solute rejection [8,9,10]. In terms of water transport performance, AqpZ exhibits a high osmotic water permeability above 10^−14^ cm^3^·s^−1^·subunit^−1^, as well as a low Arrhenius activation energy of 3.7 kcal·mol^−1^ [8,11] when incorporated into proteoliposomes/polymerosomes. Meanwhile, amphiphilic block copolymers or lipids can be self-assembled into vesicles that mimic the cell bilayer membrane, facilitating AqpZ reconstitution, and making them great candidates for preparing high-performance biomimetic membranes [12,13,14]. Novel membrane mimetics such as bicelles [15,16] and nanodiscs [17,18] are also attracting attention worldwide for membrane protein structural and functional studies besides vesicles assembled from lipid or copolymers.

Amphiphilic block copolymers provide several advantages over artificial and synthetic lipids in engineering applications, namely, high mechanical and chemical stability and design potential [19,20]. Kumar et al. demonstrated that AqpZ can be incorporated into the ABA triblock copolymer poly-(2-methyloxazoline)-*b*-poly-(dimethylsiloxane)-*b*-poly-(2-methyloxazoline) (PMOXA-*b*-PDMS-*b*-PMOXA), which is currently the most used copolymer for AqpZ reconstitution. Other copolymers typically used for the successful fabrication of vesicles include poly(butadiene)-*b*-poly(ethylene oxide) (PBD-*b*-PEO), poly(styrene)-*b*-poly(acrylic acid) (PS-*b*-PAA), poly(ethylene oxide)-*b*-poly(dimethyl siloxane)-*b*-poly(2-methyloxazoline) (PEO-*b*-PDMS-*b*-PMOXA), and poly(ethylene oxide)-*b*-poly(ethyl ethylene) (PEO-*b*-PEE) [21,22,23,24,25]. Great developments have been accomplished of styrene maleic acid (SMA)-based polymers that can form nanodiscs containing a planar lipid bilayer, which are useful to detergent-free reconstitute membrane proteins [26,27,28].

However, only a few copolymers have been found to be suitable as membrane matrixes for AqpZ reconstitution [29]. One example is the ABA triblock PMOXA-*b*-PDMS-*b*-PMOXA. One major challenge is the absence of a fundamental understanding of the interaction between AqpZ molecules and the polymer matrix materials. Hence, a systematic study of AqpZ reconstitution and vesicle formation properties of amphiphilic block copolymers with different compositions is needed to understand their composition-reconstitution relationship. This would enable a better selection of the polymeric AqpZ-reconstituted membrane matrix and broaden the usage of biomimetic membranes. In this work, vesicle formation and AqpZ reconstitution were investigated in terms of size, morphology, stability, membrane rigidity, and thermal behavior. For the first time, the composition-reconstitution relationship of the polymer matrix was investigated by DSC, and the result demonstrated that the detergent acts as the plasticizer and spacer, advancing the phase transition of the polymers and facilitating AqpZ reconstitution.

This work contributes new knowledge that will facilitate membrane matrix selection with potential copolymers that are low cost and source abundant. Furthermore, new copolymers ideal for AqpZ reconstitution can be synthesized, with great functionality and enhanced mechanical strength via tailoring the polymer properties and thereby, improving membrane performance and reducing costs in the biomimetic membranes industry.

## 2. Methods

### 2.1. Chemicals and Materials

All the block copolymers used in this work were purchased from Polymer Source (Canada, refer to Table 1 for more detailed information). The mini-extrusion set, polycarbonate (PC) membranes, and the n-dodecyl-ß-D-maltoside (DDM) detergent were purchased from Avanti Polar Lipids, Inc. 6-dodecanoyl-2-dimethylaminonaphthalene (Laurdan) are purchased from Sigma Aldrich (Singapore). The AqpZ stock solution was kindly offered by collaborators Dr. Lin Qing Song and Mr. Zhou Hu from the Department of Biological Science, National University of Singapore. All other chemicals and solvents were purchased from Sigma-Aldrich unless otherwise mentioned, Analytical reagent.

### 2.2. Copolymer Characterization

A differential scanning calorimeter (DSC, Mettler DSC822^e^) was used to characterize the thermal properties of different copolymers at a scanning rate of 10 °C/min under nitrogen purging. An amount between 5 and 10 mg of copolymer with DDM detergent at a molar ratio of 2:1, 1:1, 1:2, and 1:0 was sealed in an aluminum crucible, heated to 150 °C and kept at this temperature for 5 min to erase any prior thermal history. Then, the samples were quenched to −100 °C and heated to a temperature between 80 and 200 °C to finish a cooling-heating cycle. The temperature and power scales of the calorimeter were calibrated by melting indium, and the temperature scale was checked against melting point standards. An empty calorimetry pan was analyzed and used as the baseline for the heat-flow rate of the sample and calibration runs [30,31].

The apparent melting (T_m_) and crystallization temperatures (T_c_) were obtained from the peak temperatures of the endotherm and exotherm, respectively, in the DSC thermogram. The glass transition temperature (T_g_) was obtained as the mid-point of the change of the heat capacity, which is close to the point of inflection.

### 2.3. Vesicle Fabrication and AqpZ Reconstitution

Polymeric vesicles (polymersomes) were fabricated using a film rehydration method as reported before [32,33]. Firstly, block copolymers were dissolved in chloroform at a concentration of 10 mg/mL, then, the organic solvent was slowly removed on a rotary evaporator to form a uniform and thin film on a round-bottom flask which was further dried under strong vacuum overnight. Later, 10 mL of PBS buffer or deionized water (DI) with DDM detergent was added to allow rehydration of the thin film. Finally, a homogenous polymeric vesicular solution was obtained after gentle stirring overnight. In order to fabricate the reconstituted AqpZ lipid/polymeric vesicles (liposome/proteopolymersome), certain volume of AqpZ stock solution with a protein to polymer molar ratio of 1:200, was added in detergent during the film rehydration step and the mixture was gently agitated for a minimum of 8 h. Then, bio-beads were added stepwise into the mixture to completely remove the detergent. The resultant vesicle suspension was extruded 21 times using a polycarbonate membrane (thickness, 200 nm) to achieve a narrow size distribution. The vesicle solution was kept at 4 °C until further use. The quantification of the AqpZ reconstituted into vesicles was checked by Inductively Coupled Plasma Mass spectrometry (ICP-MS) methods according to our previous work [34].

### 2.4. Vesicle Characterization by DLS and FETEM

The size and zeta potential of the various polymeric vesicles were studied using a dynamic light scattering (DLS) Zetasizer Nano ZSP (Malvern Instrument Ltd., Malvern, UK) under different pH values in the range of pH 3 to 12. The stability of the vesicles was analyzed using a Malvern MPT-2 autotitrator, using a polymeric vesicle solution in a concentration of 0.05 mg/mL in 1 M PBS buffer. For the titration, 1 M PBS buffer solutions were prepared with pH values of 2, 7, and 12. The vesicular morphology was observed using a field emission transmission electron microscope (FETEM) JEM-2100F (JEOL, Tokyo, Japan). The samples were prepared by dropping 20 μL of 0.5 mg/mL vesicular solution on a copper grid and subsequent air drying.

### 2.5. Determination of the Arrhenius Activation Energy Using Stopped-Flow Spectrometry

In order to measure the Arrhenius activation energy of the water molecules across the polymeric vesicles, a stopped-flow experiment was carried out on a stopped-flow light scattering instrument (Chirascan Circular Dichroism Spectrometer, Applied Photophysics, Leatherhead, UK) at different temperatures for the various polymer vesicles. Briefly, the extruded vesicles were mixed with a sucrose buffer (0.6 osmol/L), which caused water efflux from vesicles that resulted in vesicle shrinkage. The size variation caused by the shrinkage was monitored and recorded in the form of an increasing signal in the light scattering analysis. The resulting curves were fit to a single-exponential rate constant (k). Then, the exponential rates (k) were plotted against the inverse of the temper/lature for the calculation of the activation energies using the Arrhenius equation.

### 2.6. Membrane Rigidity and Thermal Expansivity

A membrane probe fluorescence Laurdan (6-dodecanoyl-2-dimethylaminonaphthalene) was used to quantify membrane tension in the polymer vesicular bilayer, as reported earlier [35]. Briefly, Laurdan was added to various extruded polymersome and liposome solutions and the mixtures were incubated overnight at room temperature to allow Laurdan incorporation. The commonly used parameter generalized polarization (GP) value was calculated to reflect the chain rigidity of the lipid and polymeric bilayer, using the following equation:

$$\mathrm{GP}=\frac{I_{440}-I_{490}}{I_{440}+I_{490}}$$

where *I*_490_ is the emission peak at 490 nm associated with the liquid phase and *I*_440_ is the peak at 440 nm associated with the gel phase. High GP values typically reveal a higher chain rigidity [36,37,38].

## 3. Results and Discussion

### 3.1. Copolymer and Vesicle Characterization

The molecular weight distribution vesicle formation property, hydrophilic mass ratio, T_g_, and price of each block copolymer are listed in Table 1, as provided from the vendor. It has been previously reported that amphiphilic polymers with a proper hydrophilic mass ratio between 25 and 45% [39] have the potential to self-assemble into vesicles in suitable buffer solutions, hence, all the polymers used in this study were chosen within this range. The molecular weight of the copolymers was in the range of 7 to 13 K with relatively lower glass transition temperature, which was reported to facilitate AqpZ reconstitution [40].

**Figure 1:**
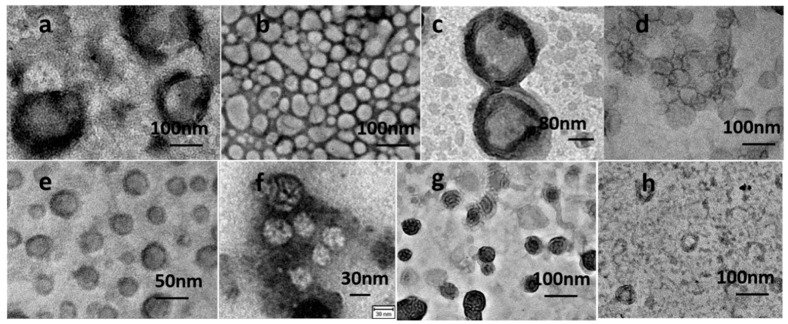
Field emission transmission electron microscope (FETEM) images of polymeric vesicles from different polymers. (**a**) Vesicles from ABANH_2_ triblock copolymers with amine ending group, (**b**) cesicles from ABAM triblock copolymers with meth acrylate ending group, (**c**) vesicles from ABAOH triblock copolymers with hydroxyl ending group, (**d**) vesicles from ABACOOH triblock copolymers with carboxyl group, (**e**) vesicles from PPO-*b*-PEO diblock copolymers, (**f**) vesicles from PBD-*b*-PEO diblock copolymers, (**g**) vesicles from PDMS-*b*-PEO, (**h**) vesicles from PS-*b*-PEO diblock copolymers.

FETEM images of the polymeric vesicles from the different copolymers are shown in Figure 1. ABA triblock copolymers with various ending groups like PDMS-*b*-PEO, PPO-*b*-PEO, and PBD-*b*-PEO can self-assemble into vesicles in DI water by the film rehydration method at room temperature as shown in Table 1 and Figure 1. However, PS-*b*-PEO, PS-*b*-PAA, and PDMS-*b*-PAA copolymers are unable to form vesicles even in the presence of detergent via the film rehydration method. This could be due to the higher glass transition temperature of the PS and PAA blocks, which is 89 and 68 °C, respectively, as shown in Table 1. Consequently, the polymer chains remain in the gel state and cannot obtain enough energy to rearrange into vesicles at room temperature [23,39].

Significant differences in the morphology and distribution of polymeric vesicles assembled from various copolymers can be observed in Figure 1. Vesicles self-assembled from ABA copolymers with methacrylate ending groups tend to be packed together like cobblestone (Figure 1b), while vesicles assembled from ABA copolymers with amine, hydroxyl, and carboxyl groups are well dispersed in the medium (Figure 1a,c,d, respectively). PDMS-*b*-PEO copolymers self-assembled into multi-lamellar vesicles while PPO-*b*-PEO and PBD-*b*-PEO copolymers assembled into the typical monolayer vesicles when rehydrated by a suitable medium (Figure 1e,f). It can be hypothesized that this phenomenon may be due to the different affinity of the ending groups with the suspending medium. For example, methacrylate groups are hydrophobic compared to other hydrophilic ending groups like amine, hydroxyl, and carboxyl group. The polymer chains can be dissolved and disperse freely for the vesicles assembled from polymers with hydrophilic ending groups due to their strong affinity with the medium (DI water). In addition, the hydrogen bond between the hydrophilic ending group and H_2_O improves the stability of the vesicles. However, vesicles assembled from ABA copolymers with methacrylate groups prefer to be packed together (Figure 1b) due to the interaction between the highly hydrophobic methacrylate group and water, hence leading to the vesicle morphology of cobblestone.

**Figure 2:**
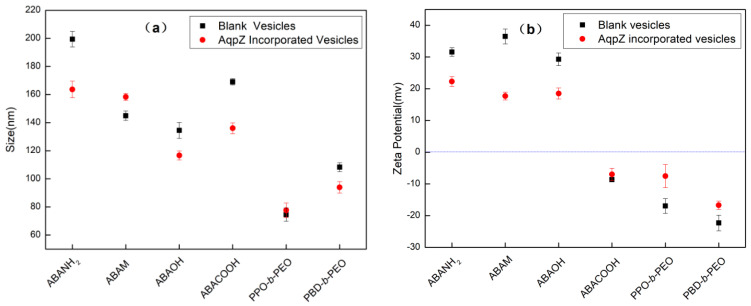
(**a**) Size and (**b**) zeta potential of various polymeric vesicles in deionized (DI)water (pH 7.4).

Vesicular size also varies broadly for the different copolymers, as shown in Figure 1 and Figure 2. ABA polymeric vesicles size ranges from 100 to 200 nm, for both the blank and the AqpZ-incorporated vesicles, while for the PPO-b-PEO and PBD-b-PEO copolymers, vesicles are in the range between 50 to 100 nm. The overall size distribution obtained from FETEM experiments is slightly smaller than that obtained from DLS measurements (Figure 2) due to differences in sample preparation and measurement. It can be observed that the zeta potential of these polymeric vesicles varies for the different copolymers, which may also be due to the different assemble behaviors caused by their chemical composition. ABA polymeric vesicles with amine, methacrylate, and hydroxyl groups presented a positive zeta potential of approximately 30 mv in the neutral medium, indicating their good stability. Meanwhile, ABA polymeric vesicles with carboxyl group and other non-ABA polymeric vesicles demonstrated negative zeta potentials under the same pH conditions. Another noteworthy observation is that the vesicles assembled from the same copolymers with or without AqpZ reconstitution showed close size and zeta potential results, demonstrating that the vesicle formation process was not affected by the protein reconstitution.

### 3.2. Arrhenius Activation Energy

**Figure 3:**
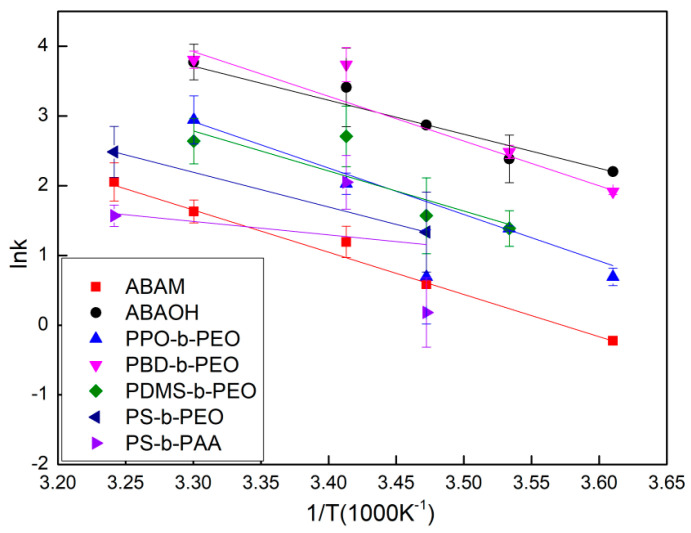
Arrhenius plots for the calculation of the activation energy for osmotic transportation of water across blank polymer vesicles.

A stopped-flow experiment was conducted at different temperatures to investigate the Arrhenius activation energy for water transport in the blank polymeric vesicles (the original stopped-flow data was supplied in Figures S1–S4). The change in *ln k* values of the blank polymeric vesicles with respect to the temperature was consistent for all the ABA triblock and other copolymers, such as PPO-*b*-PEO and PBD-*b*-PEO, exhibiting increasing *ln k* with increasing temperature (Figure 3). Higher temperatures provide higher kinetic energy to the water molecules, leading to a greater frequency, which impacts water molecule transport against the bilayer structure of the vesicles. This gives rise to a greater number of water molecules passing through the bilayer structure through the channels between the polymer chains. Additionally, there was no obvious and reproducible light scattering signal change caused by the size shrinkage of vesicles from PS-*b*-PAA and PS-*b*-PEO block copolymers, which can be explained by their poor formation due to the stiffness of the PS block. Consequently, the *k* values were not reproducible and it was not possible to obtain a *k* value at certain temperatures. This is consistent with the FETEM results in Figure 1h. Even though the diblock copolymer PDMS-*b*-PEO can be assembled into vesicles, there were no observable change in the light scattering signal due to size shrinkage, which was attributed to the increased resistance against water molecules permeating through the thick bilayer of the multi-lamellar structure (Figure 1g).

**Figure 4:**
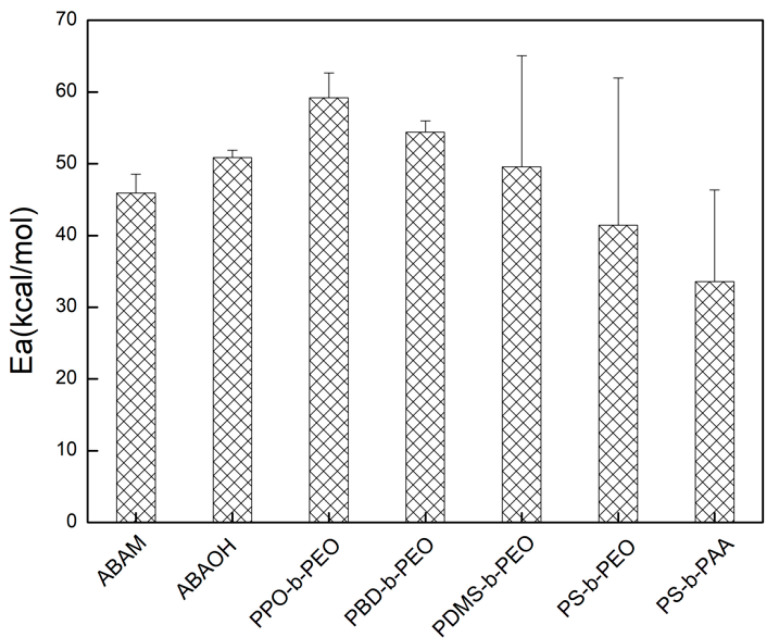
Arrhenius activation energy for osmotic transport of water across various polymeric vesicles.

The Arrhenius activation energy is calculated from the Arrhenius plot from Figure 3 and the results are shown in Figure 4. The calculated Arrhenius activation energies for various blank polymeric vesicles range from 33.6 to 59.2 kcal/mol, which are consistent with previous reported values for water transport through bilayer polymeric membranes [35]. The high Arrhenius activation energies of blank polymeric vesicles indicate that water transport through the polymer bilayer is driven by a passive diffusion mechanism. All the blank vesicles assembled from different copolymers were observed to have high Arrhenius activation energy, including the ABA triblock copolymers with the hydrophilic amine and hydroxyl ending groups, demonstrating the inherent impermeability of polymeric bilayer membranes. This result can explain why the ABA copolymers are the most ideal matrix for AqpZ reconstitution, as it is expected that water will only pass through the AqpZ water channels rather than through the bilayer polymeric membrane matrix.

### 3.3. Membrane Rigidity and Thermal Expansivity

Theoretically, GP values vary between −1.0 to +1.0; however, experimentally, they have been observed to range between 0.6 to −0.3 for lipids and copolymers. Specifically, depending on the lipid composition and temperature, GP varies from 0.3 to −0.3 for lipids in the liquid phase while the values typically range from 0.5 to 0.6 for lipids in the gel phase. Based on the literature, this method is adopted here to investigate the bilayer rigidity and thermal expansivity of the polymers [35].

**Figure 5:**
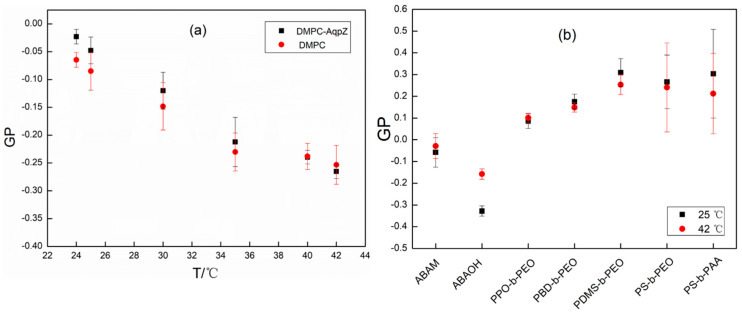
Generalized polarization (GP) for various vesicles assembled from lipid (**a**) and block polymers (**b**) at different temperatures.

First, GP in dimyristoylphosphatidylcholine (DMPC) vesicles was studied using a Laurdan probe to further verify this method and the results are shown in Figure 5a. The GP values range from 0 to −0.3 for DMPC blank vesicles (without AqpZ reconstitution) with the temperature increasing from 24 to 42 °C, which is consistent with previous reports [37]. This result indicates that the DMPC lipid vesicles were in liquid state as the phase transition temperature for pure DMPC lipid is 24 °C. The GP values for AqpZ-reconstituted DMPC vesicles were slightly higher than the blank vesicles when the temperature was lower than 35 °C, and then become slightly lower than the blank vesicles at temperatures of 40 °C and above. This result suggests that the gel to liquid transition temperature of AqpZ-reconstituted DMPC vesicles is higher than that of DMPC vesicles without AqpZ reconstitution, likely because more energy is needed for the space adjustment of AqpZ reconstitution within the vesicle bilayer. Moreover, decreasing GP values with increasing temperature indicate that the lipid vesicles tend to become more “fluidic” when enough energy is obtained at higher temperatures.

After the verification of the GP values with DMPC liposomes, polymeric vesicles were analyzed, as seen in Figure 5b. The GP values show significant differences among the various polymeric vesicles, ranging from 0.3 to −0.3 at 42 °C, which highlights the different phases for the polymeric vesicles due to their bilayer stiffness and rigidity. Moreover, the GP value did not present significant differences when the temperature increases from 25 to 42 °C, suggesting the absence of a phase transition within this temperature range for all polymeric vesicles. Another noteworthy result is that the vesicles assembled from the diblock copolymer PDMS-*b*-PEO demonstrate the highest GP values among all other polymeric vesicles. Considering the multi-lamellar morphology of PDMS-*b*-PEO vesicles from FETEM results, it can be concluded that the highest GP value is due to the increased bilayer thickness of the multilayer structure. Other vesicles demonstrate decreasing GP values in the sequence of PS-*b*-PAA > PS-*b*-PEO > PBD-*b*-PEO > PPO-*b*-PEO > ABAM > ABACOOH, which is also consistent with their formation property and the Arrhenius activation energy data. The vesicles assembled from ABA triblock copolymers hold the lowest GP values, meaning that they constitute the most fluidic vesicles at the same temperature, which makes them the most relevant candidates for AqpZ reconstitution. For the ABA copolymers, vesicles assembled from copolymers with carboxyl ending groups are more fluidic than vesicles assembled from copolymers with methacrylate ending groups. In addition, vesicles from PS-*b*-PAA and PS-*b*-PEO also showed high GP values, indicating the stiffness and rigidity of the PS block. This may be the reason for the poor vesicles’ formation performance. Altogether, these results support the hypothesis that explains the vesicle morphology as a result of the affinity of the different ending groups with the dispersed medium, as analyzed by FETEM.

### 3.4. Thermal Behavior of Detergent and Effect of the Chemical Composition by DSC

As indicated from the GP analysis, the gel to liquid phase transition of the different polymers has a significant effect on their vesicular formation and AqpZ reconstitution performance. Hence, the thermal transition behavior, including glass transition, melting, and crystallization temperatures of the different polymer matrix were further determined by DSC.

Both ABAM and ABAOH block copolymers exhibit distinct glass transition temperatures at approximately −90 °C, while the melting point of the ABAOH copolymer is slightly lower than that of the ABAM copolymer, which are −58.83 °C and −45.67 °C, respectively. This result indicates that the ABA triblock polymer is in the gel status above −90 °C and becomes more liquid above −50 °C, regardless of their ending group. The slightly higher T_m_ of the ABAM copolymer indicates that the polymer would require more energy to achieve the above-mentioned liquid status. In other words, the ABAM polymer chain is stiffer compared with that of the ABAOH polymer due to the different affinity of the hydrophobic methacrylate ending group and the hydroxy ending group with water molecules, which is consistent with the GP results. The thermal transition temperature of PPO-*b*-PEO and PBD-*b*-PEO block polymers calculated from DSC thermograms are also listed in Table 2 (the original thermograms please refer to Figure S5). The T_m_ of both PPO-*b*-PEO and PBD-*b*-PEO block polymers are much higher than that of the ABA copolymers, indicating that the PPO-*b*-PEO and PBD-*b*-PEO polymers chains are much stiffer and in the gel state. This result is also consistent with the GP analysis and the vesicular morphology results from the TEM images.

Considering the fabrication process of blank polymeric vesicles and AqpZ-reconstituted polymeric vesicles, where detergent is always involved, the effect of DDM addition at different molar ratios on the thermal transitions were also investigated by DSC (Figure S1). A melting point shift can be observed for both PPO-*b*-PEO and PBD-*b*-PEO block polymers due to the addition of DDM. Both copolymers exhibit the same trends: The melting point decreases with an increase in DDM concentration. It can be hypothesized that the DDM detergent may act as a “plasticizer”, giving the polymer chains more free volume to move within the polymer matrix, while also acting as a “spacer” which weakens the intermolecular interactions among the polymer chains. Consequently, the polymers can reach their thermal transitions at lower temperatures with the addition of DDM. This hypothesis also explains the role of the detergent on facilitating vesicle formation and AqpZ reconstitution.

### 3.5. Effect of pH on Vesicular Size and Stability

**Figure 6:**
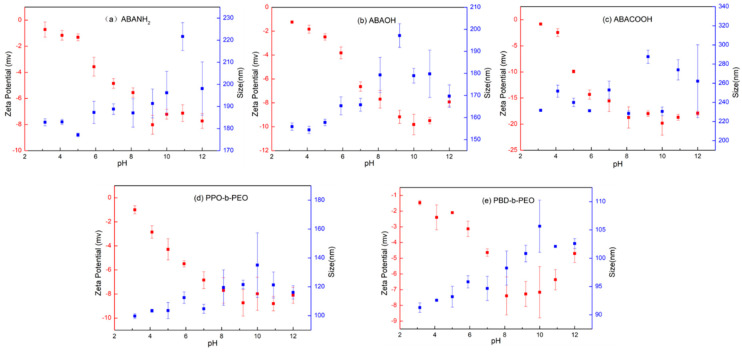
Zeta potential and size distribution of polymer vesicles self-assembled from (**a**) ABANH_2_, (**b**) ABAOH, (**c**) ABACOOH, (**d**) PPO-*b*-PEO, and (**e**) PBD-*b*-PEO under different pH values ranging from 3 to12.

Desalination processes are often performed in harsh environments, for example, at various pH levels. Hence, the effect of pH on vesicular size and stability was investigated in this work. The zeta potential and size distribution for polymeric vesicles assembled from ABA triblock copolymers with three different ending groups, and the PPO-*b*-PEO and PBD-*b*-PEO diblock copolymers were investigated under different pH values, ranging from pH 3 to 12 (Figure 6). It can be observed that the zeta potential and size distribution for all the polymeric vesicles follow the same trend: the zeta potential decreases and size slightly increases with increasing pH values. The functional hydroxyl groups present in the polymer lose their protons more easily in a basic environment, making the polymer negatively charged and decreasing their zeta potential at higher pH values.

Most of the ABA polymeric vesicles demonstrate the highest absolute zeta potential value at pH levels between 8 and 10, indicating their good stability. As analyzed from the data, it can be observed that pH has a stronger influence on the zeta potential compared with ending groups. However, it is also noteworthy that the zeta potential for polymeric vesicles assembled from ABA copolymers with carboxyl ending groups show a dramatic decrease from 0 to −20 mv (Figure 6c), while for the other ABA copolymers with hydroxyl and amine ending groups the value decreases from 0 to −10 mv (Figure 6a,b). This suggests that the ionization of the carboxyl group strongly depends on the pH of the dispersion medium. Ionization increases with increasing pH, leading to a higher absolute zeta potential value and a more stable polymeric vesicle.

Additionally, it can be observed from Figure 6 that the size distribution slightly increases from 90 to 105 nm for PBD-*b*-PEO vesicles with increasing pH value. Additionally, the zeta potential first decreases with pH values increasing from 3 to 8, then remains unchanged from 8 to 10, after which it increases with increasing pH values from 10 to 12. This indicates that the PBD-*b*-PEO vesicles are most stable under weak basic conditions from pH 8 to 10. In the future, polymers can be tuned by modification of ending groups to improve their stabilities under different pH values for greater performance on membrane protein reconstitution and structural and biophysical studies [28,41].

## 4. Conclusions

Various polymeric vesicles were self-assembled from block copolymers of different chemical composition with similar hydrophilic mass ratios through the film rehydration method. The results showed that the morphology, size, and stability among the different vesicles were greatly affected by the block copolymer composition and the ending groups. Polymers having lower glass transition temperatures or lower gel to liquid phase transition temperatures, and polymers with hydrophilic ending groups are beneficial for vesicle formation and AqpZ reconstitution. For the first time, this work revealed the composition-reconstitution relationship from a thermal perspective in terms of glass transition temperature, membrane rigidity, and expansivity. Such composition-reconstitution relationship of the polymer matrix was also studied by DSC, and it was found that the detergent acts as the plasticizer and spacer, advancing the phase transition of the polymers and facilitating AqpZ reconstitution. The results reported in this study also show the significant role of the ending groups of the polymer in the formation of polymeric vesicles. This study deepens the understanding of the mechanisms of protein incorporation based on a composition-reconstitution relationship, which enables a better selection of the copolymer matrix for enhanced biomimetic membrane performance and lower cost in desalination applications.

## Figures and Tables

**Table 1:** Physical properties and assembly behavior of the block copolymers used in this study.

Polymers	Molecular Weight Distribution	Vesicle Formation	Hydrophilic Mass Ratio	Price Per Gram (USD) *	Block T_g_ (°C)
PMOXA_1.3K_-*b*-PDMS_5K_-*b*-PMOXA_1.3K_(ABAM)	1.30	√	34.21%	450.00	-/−127/-
PPO_5.5K_-*b*-PEO_2.7K_	1.07	√	32.93%	200.00	−10/−59
PBD_5K_-*b*-PEO_2.3K_	1.03	√	31.51%	400.00	−50/−59
PDMS_5K_-*b*-PEO_2.1K_	1.20	√	29.58%	450.00	−127/−59
PDMS_8K_-*b*-PAA_5K_	1.20	×	38.46%	250.00	−127/68
PS_5K_-*b*-PEO_2K_	1.03	×	28.57%	300.00	89/−59
PS_5.2K_-*b*-PAA_4K_	1.15	×	43.48%	250.00	89/68

“√” means vesicles can be formed by the film rehydration method used here and “×” means vesicles cannot be formed by this method. * means US dollar. The ABAM here particularly refer to the ABAM block copolymers PMOXA_1.3K_-*b*-PDMS_5K_-*b*-PMOXA_1.3K_ with methoxyl ending groups. T_g_ represents the glass transition temperature of each block.

**Table 2:** T_g_, T_m_, and T_c_ of the different block copolymers calculated from their differential scanning calorimeter (DSC) plots.

Samples	T_g_	T_m_	T_c_
ABAM	−89.56	−45.67	-
ABAOH	−85.79	−58.83	-
PPO-*b*-PEO	-	40.17	19.67
PBD-*b*-PEO	−75.14	46.17	8.83

## Supplementary Materials

The following are available online at https://www.mdpi.com/2073-4360/12/9/1944/s1, Figure S1: Stop-flow results of blank vesicles from ABA copolymers with different ending groups at 37 °C; Figure S2: Stop-flow results of blank vesicles from different copolymers at 4 °C; Figure S3: Stop-flow results of AqpZ reconstituted vesicles from ABACOOH copolymers and the blank control at 4 °C; Figure S4: Stop-flow results and the exponential fitting of AqpZ reconstituted vesicles from ABAOH copolymers and the blank control at 37 °C; Figure S5: DSC thermograms of PPO-*b*-PEO (left) and PBD-*b*-PEO (right) polymers with DDM at different polymer/DDM molar ratio.
