# Supplementary material for: Investigating the Mechanisms of AquaporinZ Reconstitution through Polymeric Vesicle Composition for a Biomimetic Membrane
Source: Polymers (Basel). 2020 Aug 28;12(9):1944. doi: 10.3390/polym12091944 (PMC7565422; doi:10.3390/polym12091944)
Supplement: Supplementary file 1 [file polymers-12-01944-s001.pdf]

Supporting Information:

1. Expression and purification of Aquaporin-Z

The modified pET plasmid containing AqpZ gene with N-terminal 6x his affinity tag was transformed into E. coli strain BL21 star (DE3) (Invitrogen, Carlsbad, CA). Cells from a single colony were inoculated in LB medium with 100 µg/ml ampicillin and grown overnight at 37 °C. The overnight cultures were diluted 100 fold into fresh LB medium and propagated to an $A_{600\ nm}$ of 1.2–1.5. The culture was induced with 1 mM isopropyl b-D-1-thiogalactopyranoside and grown at 37 °C for 2 h before harvesting.

The cells of 1L culture was harvest by centrifugation at 6000 rpm for 15 min and resuspended in 10 mL of lysis buffer containing 20 mM Tris–HCl (pH 8.0), 100 mM NaCl, 1 mM $MgSO_4$, 1 mM phenylmethanesulfonyl fluoride and 0.1 mg/mL deoxyribonuclease I. Cell resuspension was subjected to sonication and the lysate was centrifuged at 10,000 g for 30 min to remove the insoluble material. Membrane fraction was recovered from the supernatant by centrifugation at 140, 000 g for 1 h.

For Aqp extraction, the membrane fraction was resuspended with solubilization buffer of 1 % n-dodecyl-beta-maltoside (DDM) in a buffer containing 20 mM Tris–HCl (pH 8.0), 100 mM NaCl and incubated overnight at 4 °C. Insoluble material was pelleted by 45 min centrifugation at 140, 000 g. The DDM solubilized AqpZ was bound to cobalt

resin by gentle shaking at 4 °C for 2 h in the presence of 5 mM imidazole. The protein bound cobalt resin was washed with 10 column volumes of buffer containing 20 mM Tris–HCl (pH 8.0), 100 mM NaCl, 10 mM imidazole and 0.2% DDM. AqpZ was then eluted with washing buffer supplemented with 150 mM imidazole.

2. The molecular structure of copolymers

$PMOXA_{1.3K}$-*b*-$PDMS_{5K}$-*b*-$PMOXA_{1.3K}$(ABAM) ABA triblock copolymers:

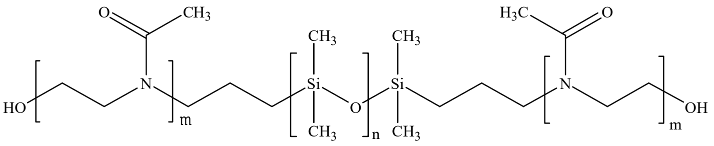

$PEO_{2.7K}$-*b*-$PPO_{5.5K}$:

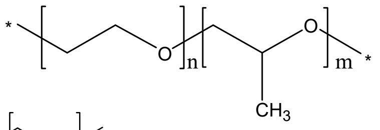

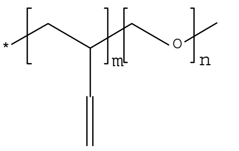

PBD_{5K}-*b*-$PEO_{2.3K}$:

$PDMS_{5K}$-*b*-$PEO_{2.1K}$:

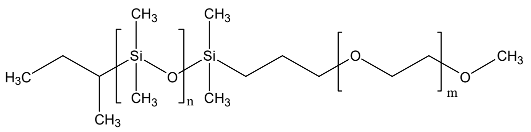

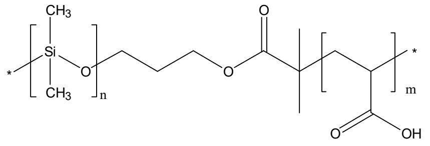

$PDMS_{8K}$-*b*-PAA_{5K}:

PS_{5K}-*b*-PEO_{2K}:

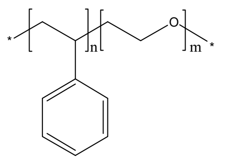

$PS_{5.2K}$-*b*-PAA_{4K}:

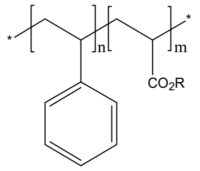

3. Stop-flow dynamic curves of various copolymers:

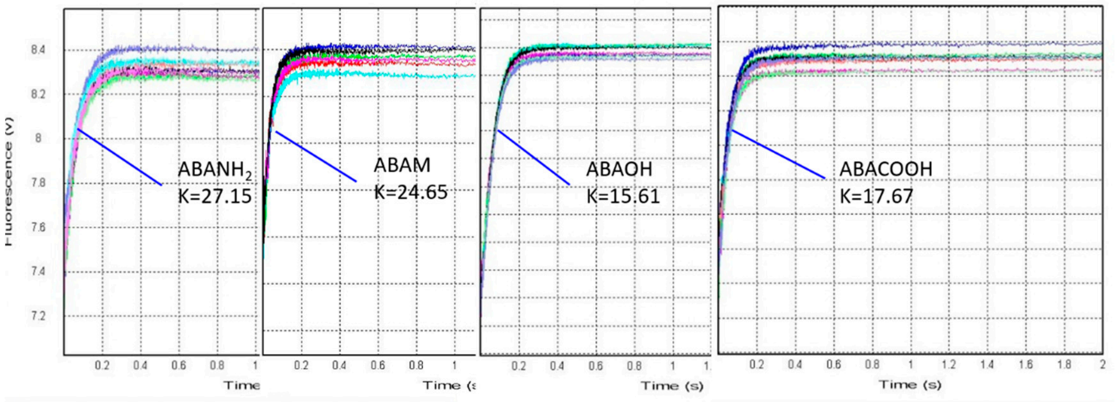

Figure S1: Stop-flow results of blank vesicles from ABA copolymers with different ending groups at 37 °C

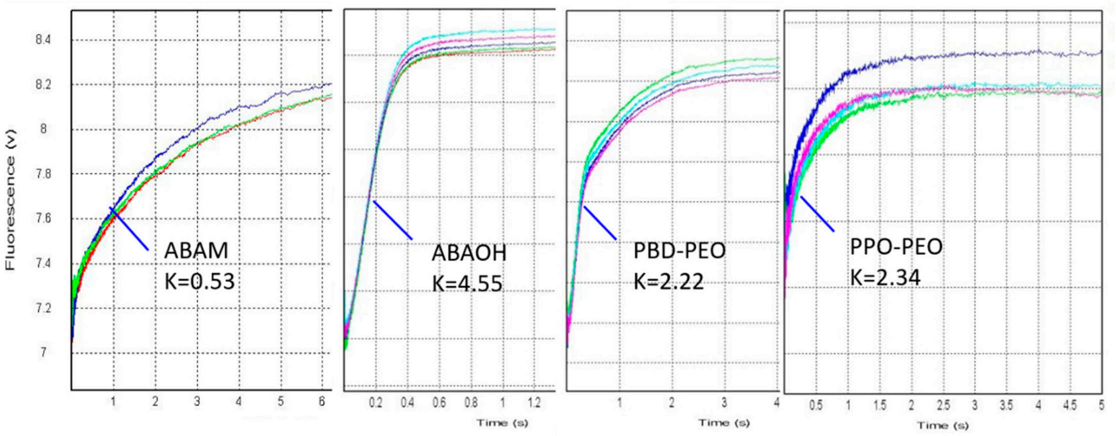

Figure S2: Stop-flow results of blank vesicles from different copolymers at 4 °C

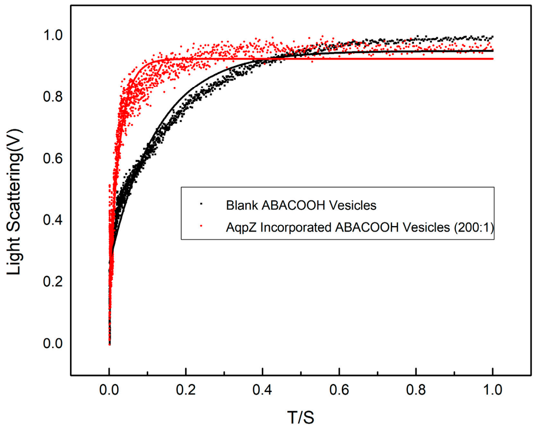

Figure S3: Stop-flow results of AqpZ reconstituted vesicles from ABACOOH copolymers and the blank control at 4 °C.

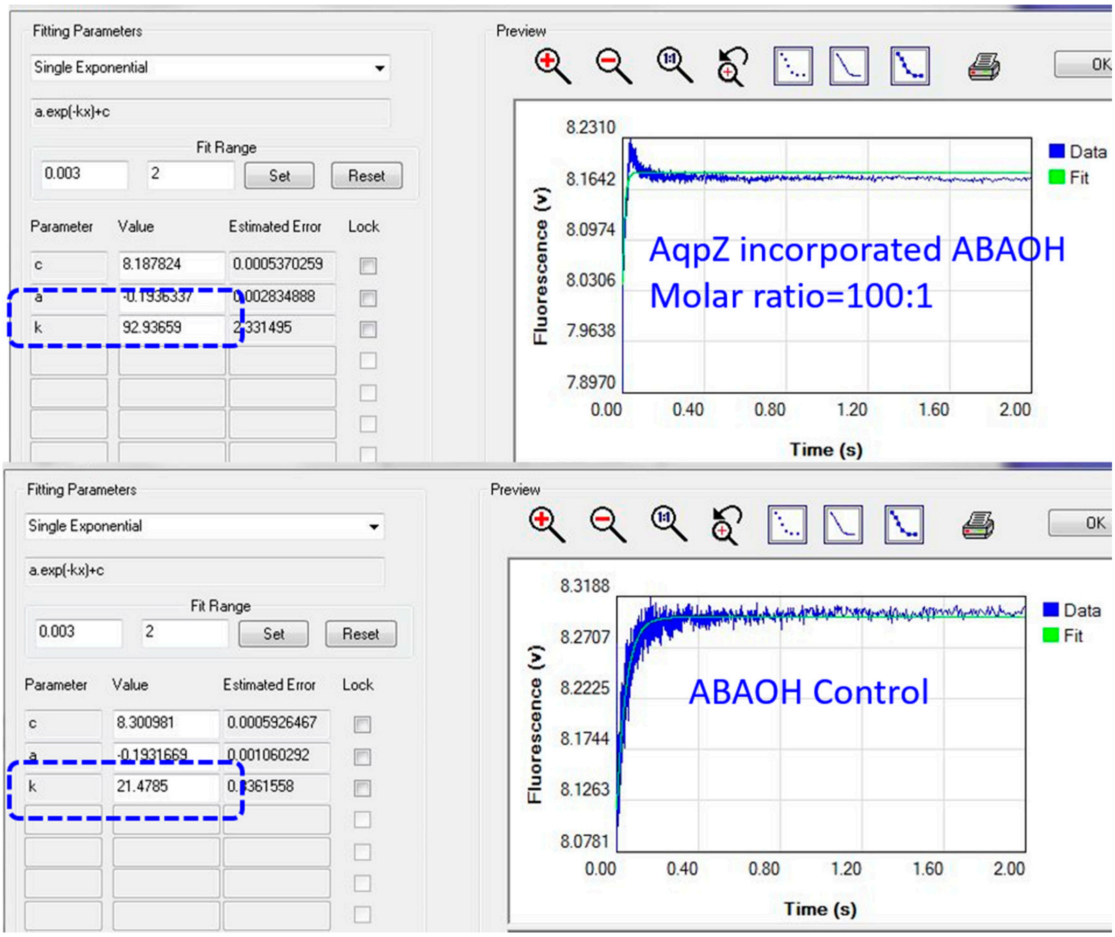

Figure S4: Stop-flow results and the exponential fitting of AqpZ reconstituted vesicles from ABAOH copolymers and the blank control at 37 °C.

4. DSC thermograms of PPO-b-PEO (left) and PBD-b-PEO (right) polymers with DDM at different polymer/DDM molar ratio.

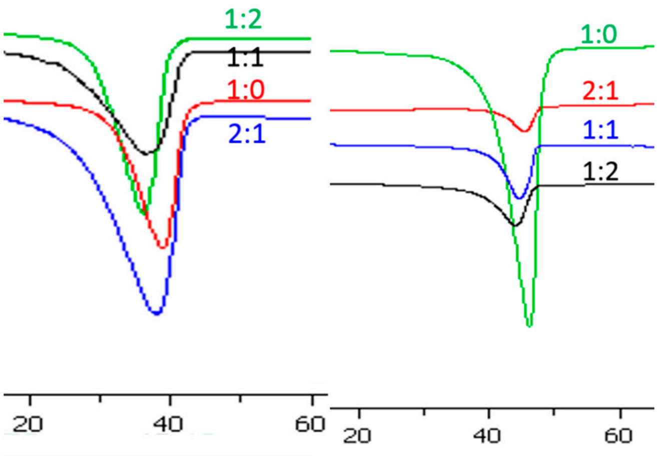

Figure S5: DSC thermograms of PPO-b-PEO (left) and PBD-b-PEO (right) polymers with DDM at different polymer/DDM molar ratio.
